# Retinal layer segmentation in a cohort of healthy children via optical coherence tomography

**DOI:** 10.1371/journal.pone.0276958

**Published:** 2022-11-03

**Authors:** Anna-Katharina Runge, Jana Remlinger, Mathias Abegg, Thomas Ferrazzini, Dominik Brügger, Katharina Weigt-Usinger, Thomas Lücke, Ralf Gold, Anke Salmen

**Affiliations:** 1 Department of Neurology, Inselspital, Bern University Hospital, and Department of Biomedical Research, University of Bern, Bern, Switzerland; 2 Department of Ophthalmology, Inselspital, Bern University Hospital, University of Bern, Bern, Switzerland; 3 Graduate School for Cellular and Biomedical Sciences, University of Bern, Bern, Switzerland; 4 Department of Neuropaediatrics, St. Josef-Hospital, Ruhr-University Bochum, Bochum, Germany; 5 Department of Neurology, St. Josef-Hospital, Ruhr-University Bochum, Bochum, Germany; Charite Universitatsmedizin Berlin, GERMANY

## Abstract

**Background:**

High-resolution optical coherence tomography (OCT) allows the detection of macular pathology and involvement of the optic nerve in a wide spectrum of diseases. For the differentiation of diseased and healthy status, normal values of retinal layer segmentation are critical. Yet, normative values mostly cover adult populations with only sparse data for paediatric cohorts. We present data of retinal layer characteristics via OCT in a healthy paediatric cohort.

**Methods:**

This prospective cross-sectional study screened 75 healthy children (male = 42, female = 33, range 4–17 years) without visual problems. OCT was performed with a peripapillary ring and macula scan protocol to determine paediatric normative values for routine parameters (peripapillary retinal nerve fibre layer thickness (pRNFL), total macular volume (TMV), macular retinal thickness (RT)). The macula scan (6mm grid) was segmented using the device-inherent automated segmentation software (Heidelberg Eye Explorer) for retinal layers: RNFL, ganglion cell layer (GCL), inner plexiform layer (IPL), inner nuclear layer (INL), outer plexiform layer (OPL), outer nuclear layer (ONL) in 9 segments each and mean of the 9 segments.

**Results:**

We obtained OCT data of 72 children with mean age 12.49 years (standard deviation, SD, 2.18; minimum 3.93). Mean global pRNFL was 102.20 μm (SD 8.24), mean TMV 8.81 mm^3^ (0.30) and mean RT (all segments) 318.22 μm (10.19). Segmented macular retinal layer thicknesses (mean of all segments) were: RNFL 27.67 μm (2.14), GCL 41.94 μm (2.50), IPL 34.97 μm (2.10), INL 35.18 μm (2.15), OPL 29.06 μm (2.24), ONL 68.35 μm (6.20).

**Conclusion:**

The OCT is a useful non-invasive imaging technique for the examination of the retina in children with short duration, high imaging resolution and no known adverse effects. Normative values may serve as a comparator for different neuropaediatric disorders and are first presented with this study using an up-to-date and standardized OCT imaging technique.

## Introduction

In the course of the last 30 years, optical coherence tomography (OCT) has become the single most important imaging modality in ophthalmology [1]. The more recent advent of high resolution OCT and automatic segmentation of retinal layers further increased the sensitivity and specificity to detect important diseases such as macular pathology and diseases of the optic nerve. For the latter, the analysis of macular ganglion cell layer thickness shows benefits as compared to the more standard analysis of the peripapillary nerve fibre layer [2]. The separation of healthy status from disease may be on a morphological basis, but is often defined by a deviation of parametric values from normal values. In the context of OCT, the thickness of peripapillary retinal nerve fibre layer thickness (pRNFL) for example allows to identify on a statistical basis patients with optic neuropathy or disc swelling. Such normative data may thus be critical in the detection of diseases. However, normative data, especially for macular layer segmentation, are not available for the population of persons younger than 18 years. As OCT is easy to use and has no adverse effects such as radiation, it is a feasible tool in the examination of children with (neuro-) ophthalmological conditions.

As retinal thickness decreases with age in healthy adults [3–7], it is uncertain whether adult normative values can be extrapolated to children. The evolution of retinal thickness during childhood is so far unclear. Previous studies have only examined standard OCT measures (pRNFL) and/or macular volume/thickness) in healthy children with varying OCT techniques [8–12]. Only one study describes normative values for different retinal layers after segmentation, yet, using a custom-made software and may thus be difficult to transfer to other devices [13].

This study aims to examine a healthy paediatric population to establish normative values of standard OCT measures (pRNFL, total macular volume (TMV), macular retinal thickness (RT)) and the segmented macular retinal layers using the device-inherent standardized software.

## Methods

### Study design and participants

Seventy-five children were recruited from school classes and nurseries via the Neuropaediatric department of the Clinic for paediatric and adolescent medicine in Bochum in a cooperation with the department of Neurology, St. Josef-Hospital, Ruhr-University Bochum. Informed consent was obtained from at least one parent/legal guardian and the children themselves. The parents were given a questionnaire to detect co-morbidities. Boys and girls under 18 years of age and without visual/ocular diseases were included in the study. Axial length measurement or other ophthalmological evaluations were not performed.

### In- and exclusion criteria

Of the 75 screened, 3 children had to be excluded from the study: 2 children due to premature birth and 1 child because of high myopia. In addition, 2 children were found with concomitant medication. This was not deemed to influence the results by the principal investigators. The exclusion criteria are shown in Table 1.

**Table 1:** Exclusion criteria.

**Exclusion criteria**	
**Ophthalmological diseases**	• Severe myopia (more than -6.0 dioptre) • Optic disc drusen • Optic nerve head drusen • Cataract • Glaucoma • Pronounced vitreous opacity / mouches volantes • Known infection with toxoplasmosis in medical history • Retinitis / chorioretinitis in medical history
**Immunological diseases**	
**Oncological diseases**	
**Neurological diseases involving the central nervous system**	• neuroimmunological or neurodegenerative disorders such as juvenile Multiple Sclerosis or Morbus Huntington
**Hereditary or acquired metabolic diseases with putative impact on the central nervous system**	• glycogenosis • diabetes mellitus type 1 • juvenile syndromes of epilepsy • pseudotumor cerebri
**Premature delivery**	
**Regular medication**	• anticonvulsive drugs • carbonic anhydrase inhibitors
**Substance abuse**	
**Known steroid intake during the last eight weeks preceding the examination**	
**HIV-infection or other immunodeficiency syndromes**	
**Signs of head injury or severe traumatic brain injury in the previous medical history**	

Abbreviations: HIV–human immunodeficiency virus

### OCT

The examination was done using a spectral domain OCT (SD-OCT) (Spectralis®-OCT, Heidelberg Engineering, Heidelberg, Germany).

Two types of scans were used within this study: a peripapillary scan and a macular scan.

**Fig 1:**
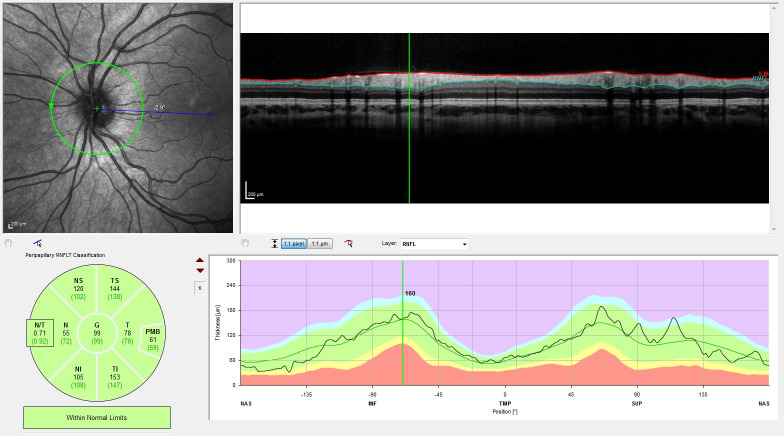
Exemplary peripapillary scan, oculus sinister (OS). Upper left: fundus image, upper right: OCT scan of retinal layers, lower left: RNFL values per sector (in μm), lower right: RNFL values over the whole OCT scan with integration in normative percentiles (as set by the device for adults) and coloured areas to indicate values outside the normal range. Abbreviations: OCT–optical coherence tomography, RNFL–retinal nerve fibre layer.

The peripapillary scan can be analysed using the thickness profile, which gives the cross-sectional view of the retina in a circle around the papilla (Fig 1). The resolution mode for the peripapillary scan was high resolution. The scan pattern used was 1 B-scan with a diameter of 3.6mm. The quality parameters were set to be automatic real-time (ART) ≥ 30 (max. 100) and a quality ≥ 25. Seven sectors are given for this ring scan: global (G), nasal (N), temporal (T), nasal-superior (NS), nasal-inferior (NI), temporal-superior (TS) and temporal-inferior (TI). The papillo-macular bundle (PMB) is separately measured. N/T gives the ratio of the nasal and temporal sector. The automated pRNFL measurement compares the measured values to deposited adult normative values.

**Fig 2:**
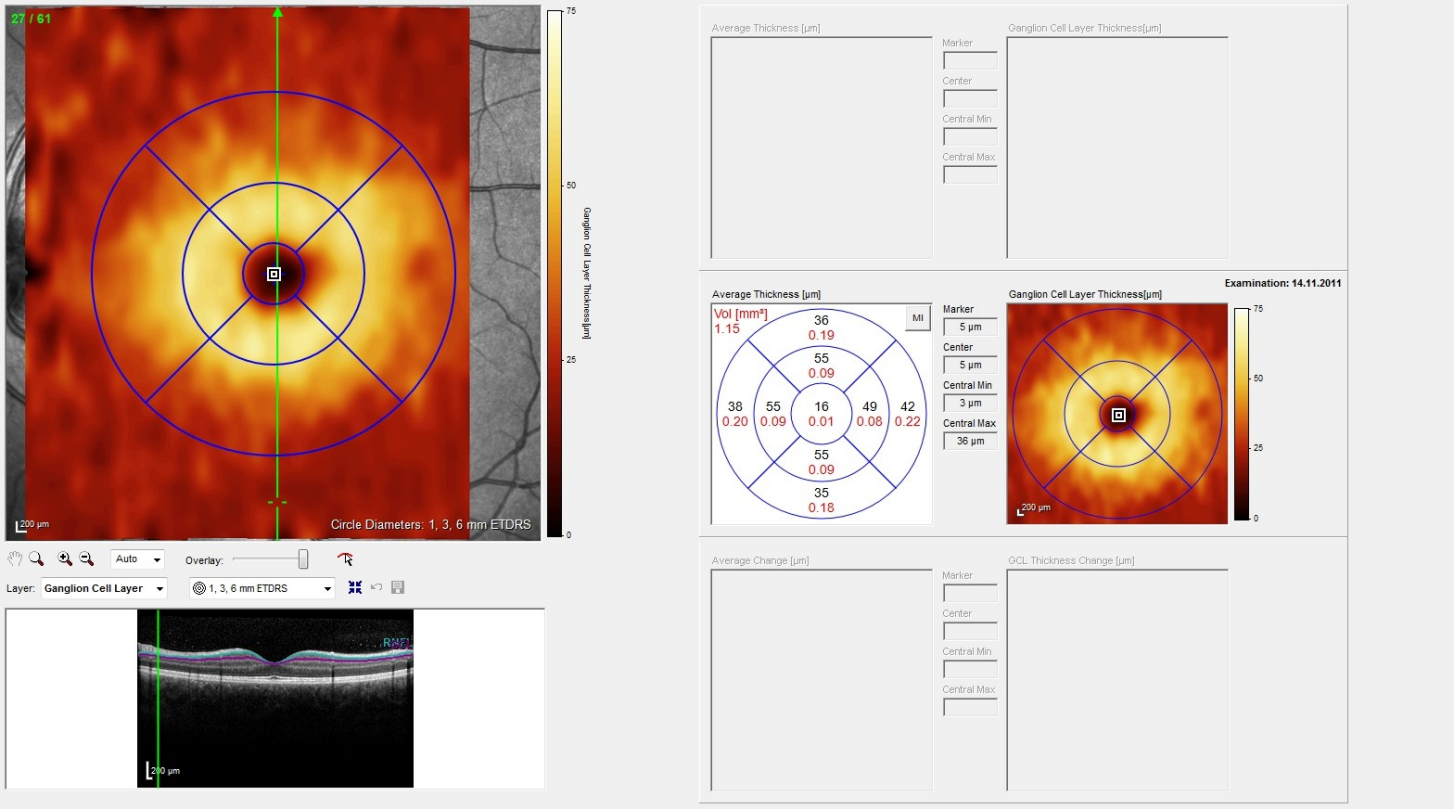
Macular scan, oculus sinister (OS). Upper left: fundus image with colours coding for GCL, lower left: OCT scan of retinal layers, right: GCL values per sector and coded by colours. Abbreviations: OCT–optical coherence tomography, GCL–ganglion cell layer.

The macular scan is shown in Fig 2. The resolution mode for the macular scan was a high resolution mode. The scan pattern used was 61 B-scans in a distance of 124µm and with an early treatment diabetic retinopathy study (ETDRS) grid size of 1, 3 and 6mm (radius). The quality parameters were set to be ART ≥ 11 and a quality ≥ 25. The fovea is centred (centre point) with two surrounding circles. Each circle is divided into 4 sectors: nasal (N1 / N2), temporal (T1 / T2), superior (S1 / S2), inferior (I1 / I2), 1 representing the inner, 2 representing the outer circle. The sector surrounding the centre point with 1mm diameter is called C0.

**Fig 3:**
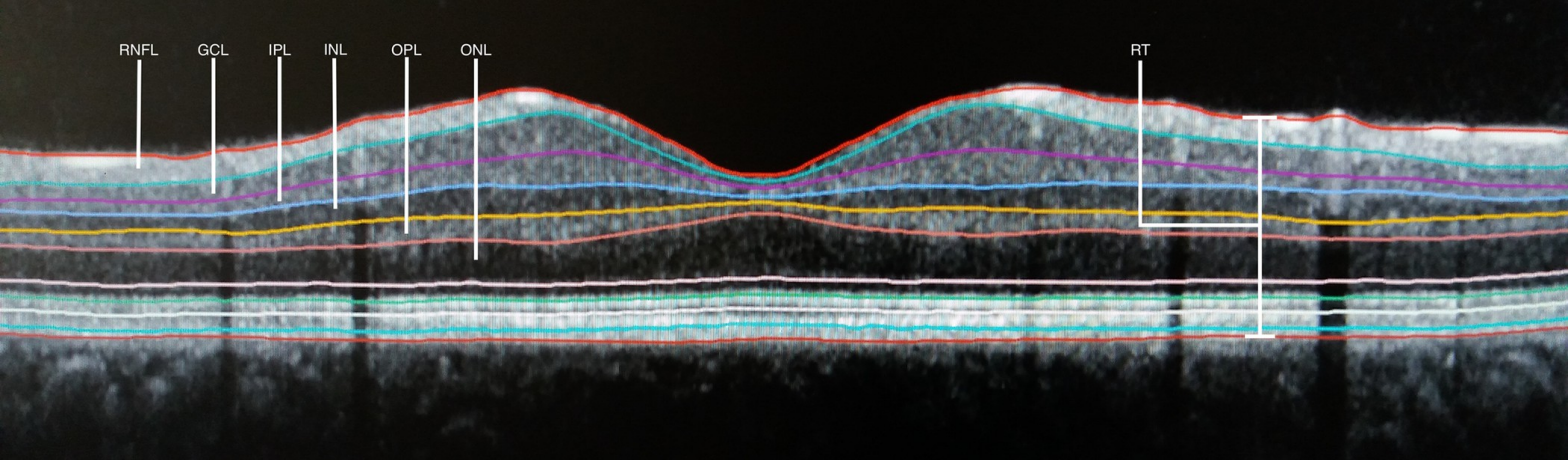
Cross-sectional representation of the macula showing the segmentation of retinal layers. From top to bottom: Retinal nerve fibre layer (RNFL), ganglion cell layer (GCL), inner plexiform layer (IPL), inner nuclear layer (INL), outer plexiform layer (OPL), outer nuclear layer (ONL); retinal thickness (RT).

pRNFL, TMV and RT were provided by the software. For macular retinal layer segmentation, the segmentation software (Segmentation Batch, Heidelberg Eye Explorer) was used segmenting all layers of the retina automatically (Fig 3). The internal scan quality measure was defined to be ≥25 to be included in the analysis. Scans which did not fully display the retina were excluded by the software and did not undergo the analysis.

Manual correction was deliberately abstained from in our study to only include results that the segmentation software determined using a segmentation algorithm and to avoid potential bias caused by manual adaptation. Scans were checked for errors occurring during the segmentation process: Scans with minor inaccuracies not remarkably affecting the results were not manually adapted, but still included; scans with major inaccuracies were excluded from the study.

Normative values for the inner retinal layers were analysed for a total volume of the 6mm grid in mm^3^, for all 9 sectors (C0, N1/2, T1/2, S1/2, I1/2) and a mean of these sectors in µm: 1) RNFL, 2) GCL, 3) IPL, 4) INL, 5) OPL, 6) ONL.

### Statistics

All data were exported from the Heidelberg Eye Explorer to Microsoft Excel, Version 16.35 for Mac. All statistical analyses were performed using GraphPad Prism Software, Version 8.0.1 for Windows, GraphPad Software, Inc., La Jolla, California, USA. To account for a potential bias due to inter-eye correlation in individuals with two eyes included in the analyse, an outlier identification was performed using the ROUT method, based on the False Discovery Rate (FDR), with a Q set to 1%. Outliers were present in 37 individuals vs. 35 individuals without any outlier (102 detected outliers in total of 14’740 values). Using Fisher’s exact test, no different distribution of participants with one or both eyes included in the analysis for the presence of any outlier was detected (p = 0.57). Results are thus presented using values of both eyes whenever available. The full outlier analysis is given in the open data repository (https://doi.org/10.48620/68).

### Approval of ethics committee

The study has been approved by the ethics committee of the medical faculty of Ruhr-University Bochum with the registration number 3952–11 and complies with the tenets of the Declaration of Helsinki. Written informed consent was obtained from at least one parent/legal guardian and the individual participants included in the study.

Due to the change of the principal investigator, the anonymised evaluation of the data was continued in a new centre as deemed appropriate by the responsible committee (Cantonal Ethics Committee Bern). All data were anonymized prior to any analysis.

## Results

### Cohort characteristics

Demographic data of the cohort are shown in Table 2. The age distribution of the cohort is presented in detail in Table 3.

**Table 2:** Summary of participant characteristics.

Characteristics		
Screened participants		n = 75
Excluded participants	before OCT scan	n = 3 (myopia (n = 1), preterm birth (n = 2))
Included participants	final cohort	n = 72 (144 eyes)
Age [years]	mean, SD	12.49, 2.18
	median (range)	12.38 (3.93–17.00)
Sex [n (%)]	female	32 (44)
	male	40 (56)
Comedication [n (%)]	total	2 (3)
	concomitant medication used	amlodipine (n = 1)
		salbutamol (n = 1)
Evaluable peripapillary RNFL scans [n]	all eyes	128
	left eyes	67
	right eyes	61
Evaluable macular scans for TMV / RT [n]	all eyes	114
	left eyes	57
	right eyes	57
Evaluable macular scans for layer segmentation [n]	all eyes	126
	left eyes	65
	right eyes	61

Abbreviations: OCT–optical coherence tomography, RNFL–retinal nerve fibre layer, RT–retinal thickness SD–standard deviation, TMV–total macular volume.

**Table 3:** Age distribution of the final cohort.

Age in years	Number of participants of this age
4	1
5	0
6	0
7	1
8	1
9	0
10	4
11	16
12	14
13	17
14	4
15	7
16	6
17	1

### OCT measurements

#### Peripapillary retinal nerve fibre layer

Data of the pRNFL measurement are shown in Table 4.

**Table 4:** Peripapillary RNFL measurements.

	n	Mean	Standard deviation	95%-confidence interval	
Quality	128	35.55	4.64	34.74	36.36
ART	128	94.69	12.98	92.42	97.00
pRNFL—G [µm]	128	102.20	8.24	100.75	103.64
pRNFL—PMB [µm]	128	56.84	8.06	55.43	58.25
pRNFL—N/T [ratio]	128	0.98	0.28	0.93	1.03
pRNFL—NS [µm]	128	114.78	19.10	111.44	118.12
pRNFL—N [µm]	128	72.24	14.59	69.69	74.79
pRNFL—NI [µm]	128	112.14	22.38	108.23	116.05
pRNFL—TI [µm]	128	149.49	15.82	146.73	152.26
pRNFL—T [µm]	128	74.70	10.19	72.91	76.48
pRNFL—TS [µm]	128	146.22	15.85	143.45	148.99

Abbreviations: ART–automatic real-time, G–global, N–nasal, NI–nasal-inferior, NS–nasal-superior, N/T–nasal/temporal ratio, PMB–papillo-macular bundle, pRNFL–peripapillary retinal nerve fibre layer.

An explorative correlation analysis of age vs. pRNFL (G) was not significant in the small age range of this study (Pearson’s r 0.07, p-value 0.46).

#### Macular scans

Automated TMV and RT as well as segmentation data were acquired on the same scans (mean quality 31.85, SD 3.23; mean ART 14.64, SD 6.44).

#### Total macular volume and retinal thickness

TMV and RT data are shown in Table 5 as generated by the standard OCT algorithm of the macular scan. Corresponding values gathered with the segmentation software differ only slightly (data given in https://doi.org/10.48620/68).

**Table 5:** TMV and RT analysis of macular scan sectors.

	n	Mean	Standard deviation	95%-confidence interval	
TMV [mm^3^]	114	8.81	0.30	8.75	8.86
RT—C0 [µm]	114	273.70	17.37	270.48	276.92
RT—N1 [µm]	114	340.92	14.61	338.21	343.63
RT—N2 [µm]	114	307.16	20.58	303.34	310.98
RT—S1 [µm]	114	348.61	11.41	346.49	350.72
RT—S2 [µm]	114	305.15	11.71	302.98	307.32
RT—T1 [µm]	114	342.53	13.82	339.96	345.09
RT—T2 [µm]	114	305.54	20.46	301.75	309.34
RT—I1 [µm]	114	345.23	11.78	343.04	347.41
RT—I2 [µm]	114	295.12	11.73	292.95	297.30
RT—all sectors [µm]	114	318.22	10.19	316.33	320.11

Abbreviations: RT–retinal thickness, TMV–total macular volume.

An explorative correlation analysis of age vs. TMV was not significant in the small age range of this study (Pearson’s r 0.14, p-value 0.14).

#### Segmentation data

Inner retinal layer volumes of the 6mm grid and thicknesses for RNFL, GCL, IPL, INL, OPL and ONL are shown in Tables 6–11.

**Table 6:** RNFL segmentation.

RNFL					
	n	Mean	Standard deviation	95%-confidence interval	
Total Volume [mm^3^]	126	0.93	0.08	0.92	0.94
C0 [µm]	126	12.94	1.83	12.62	13.27
N1 [µm]	126	21.45	2.20	21.06	21.84
N2 [µm]	126	51.42	5.79	50.40	52.44
S1 [µm]	126	25.27	2.75	24.78	25.75
S2 [µm]	126	37.80	4.33	37.04	38.56
T1 [µm]	126	16.68	1.04	16.50	16.87
T2 [µm]	126	17.84	1.05	17.65	18.03
I1 [µm]	126	25.60	2.79	25.11	26.10
I2 [µm]	126	40.04	5.18	39.13	40.95
All sectors [µm]	126	27.67	2.14	27.30	28.05

Abbreviations: RNFL–retinal nerve fibre layer

**Table 7:** GCL segmentation.

GCL					
	n	Mean	Standard deviation	95%-confidence interval	
Total Volume [mm^3^]	126	1.12	0.07	1.11	1.14
C0 [µm]	126	18.56	5.43	17.60	19.51
N1 [µm]	126	54.55	4.22	53.80	55.29
N2 [µm]	126	39.02	3.38	38.43	39.62
S1 [µm]	126	54.31	3.67	53.66	54.96
S2 [µm]	126	34.79	2.78	34.30	35.28
T1 [µm]	126	50.47	3.84	49.79	51.15
T2 [µm]	126	37.98	3.83	37.31	38.66
I1 [µm]	126	53.58	3.69	52.93	54.23
I2 [µm]	126	34.21	2.84	33.71	34.72
All sectors [µm]	126	41.94	2.50	41.50	42.28

Abbreviations: GCL–ganglion cell layer

**Table 8:** IPL segmentation.

IPL					
	n	Mean	Standard deviation	95%-confidence interval	
Total Volume [mm^3^]	126	0.92	0.06	0.91	0.93
C0 [µm]	126	23.71	4.08	23.00	24.43
N1 [µm]	126	43.34	2.95	42.82	43.86
N2 [µm]	126	30.66	2.76	30.17	31.15
S1 [µm]	126	43.33	2.97	42.80	43.85
S2 [µm]	126	28.20	2.36	27.78	28.61
T1 [µm]	126	41.46	3.01	40.93	41.99
T2 [µm]	126	33.04	2.52	32.60	33.48
I1 [µm]	126	43.36	2.88	42.85	43.87
I2 [µm]	126	27.68	2.44	27.25	28.11
All sectors [µm]	126	34.97	2.10	34.61	35.34

Abbreviations: IPL–inner plexiform layer

**Table 9:** INL segmentation.

INL					
	n	Mean	Standard deviation	95%-confidence interval	
Total Volume [mm^3^]	126	0.99	0.06	0.98	1.00
C0 [µm]	126	19.14	4.63	18.32	19.95
N1 [µm]	126	40.56	3.65	39.91	41.20
N2 [µm]	126	34.89	2.70	34.41	35.37
S1 [µm]	126	41.43	3.52	40.81	42.05
S2 [µm]	126	34.01	2.45	33.58	34.44
T1 [µm]	126	38.58	3.14	38.03	39.13
T2 [µm]	126	34.46	2.51	34.02	34.90
I1 [µm]	126	39.91	3.28	39.33	40.48
I2 [µm]	126	33.68	2.28	33.27	34.08
All sectors [µm]	126	35.18	2.15	34.80	35.56

Abbreviations: INL–inner nuclear layer

**Table 10:** OPL segmentation.

OPL					
	n	Mean	Standard deviation	95%-confidence interval	
Total Volume [mm^3^]	126	0.79	0.05	0.78	0.80
C0 [µm]	126	24.80	4.11	24.08	25.53
N1 [µm]	126	32.12	8.42	30.63	33.60
N2 [µm]	126	27.40	3.80	26.73	28.07
S1 [µm]	126	33.46	4.90	32.60	34.33
S2 [µm]	126	26.77	1.91	26.43	27.11
T1 [µm]	126	32.49	7.11	31.24	33.75
T2 [µm]	126	26.90	2.70	26.42	27.37
I1 [µm]	126	31.20	4.07	30.48	31.92
I2 [µm]	126	26.41	1.95	26.06	26.75
All sectors [µm]	126	29.06	2.24	28.67	29.46

Abbreviations: OPL–outer plexiform layer

**Table 11:** ONL segmentation.

ONL					
	n	Mean	Standard deviation	95%-confidence interval	
Total Volume [mm^3^]	126	1.80	0.16	1.77	1.83
C0 [µm]	126	88.31	10.01	86.55	90.07
N1 [µm]	126	74.29	11.38	72.28	76.29
N2 [µm]	126	61.16	7.14	59.90	62.42
S1 [µm]	126	69.06	8.38	67.58	70.53
S2 [µm]	126	64.00	5.92	62.96	65.04
T1 [µm]	126	71.25	9.23	69.62	72.87
T2 [µm]	126	60.91	5.80	59.89	61.93
I1 [µm]	126	69.41	7.72	68.04	70.77
I2 [µm]	126	56.77	5.73	55.76	57.78
All sectors [µm]	126	68.35	6.20	67.26	69.44

Abbreviations: ONL–outer nuclear layer

## Discussion

The OCT has become an important diagnostic tool to acquire *in vivo* images of the retina at a spatial resolution in the range of microns [14]. We set out to determine normative values in a healthy paediatric cohort for future use in different paediatric disorders.

In our study, the examination process was quick, and no major difficulties occurred. One main restriction of the OCT that we hypothesized was the age of the children, because a young child might not sit quiet during the required time. However, we observed an overall good compliance. This will alleviate early detection of ocular diseases such as glaucoma by OCT with consecutive earlier treatment initiation and limitation of disease progression [15–17].

In other paediatric populations with healthy eyes, OCT proved good reproducibility [13, 18]. Our results additionally demonstrate feasibility in a routine clinical setting.

Our study provides normative values of healthy children that can be used as a reference in studies of specific disorders or in clinical practice. The OCT protocols used here are standard pre-set protocols as detailed above. This allows the use of our data in different settings with good comparability.

Comparison of previous studies evaluating children to our study is limited. Only few evaluate healthy children and report standard OCT parameters (pRNFL, TMV, RT) [8–11]. Some studies only focus on few retinal layers [18, 19]. To our knowledge, only one study with complete segmentation data has been published before, but used a custom software which may hamper broader clinical applicability and comparability [13]. We assume that different segmentation algorithms define the border between layers slightly differently and the thickness values derived from distinct layers may not be directly comparable. This is a limitation of the current study, as our findings are most likely only valid for examinations done with a Heidelberg Spectralis system, one of the most commonly used devices, but still a clear issue in the comparison of different OCT studies.

Within previous studies, different OCT protocols have been used or are sometimes not precisely described which may lead to different results [3, 12]. Comparability of the data is thus limited due to a lack of precise information of the machine used, the areas analysed or the diameters of the sectors [12].

Most and best researched in children are values for pRNFL. The mean global pRNFL measured during our study is comparable to other cohorts including adults [4, 5, 20] and children [21, 22]. Half of the other studies report slightly higher mean pRNFL values [10, 13, 23–25] and the other half slightly lower mean pRNFL values [8, 9, 11, 20, 26], respectively. Most of these studies were performed using SD-OCT as in our study, some using Stratus OCT [9, 10, 24].

TMV could not be compared due to previously mentioned discrepancies (as for example the use of OCT-3 [16], Cirrus OCT [27] or a grid of 3.5mm [21]).

The age distribution in our analysis with most participants between 10 and 16 years might limit transferability of the data to very young children. We did not detect a correlation of age and pRNFL or TMV, yet, our cohort was not powered to examine age effects and this might be attributed to a narrow age range and not hold true for younger children. This is why we refrained from additional correlation analyses for all retinal layers with age or other parameters. Still, in one study, the influence of an age-related decrease in RNFL/retinal thickness seemed to be negligible from the age of 4 years and older [3] whereas another study reported an increase of the macular thickness with age for the central subfield [13] in a cohort of comparable age to ours. Correlations of some OCT parameters with age seem to be biologically reasonable as a study found evidence of progressive foveal development in children [7]. It might be interesting to longitudinally observe the development of the eyes of children from birth on as the current data remains inconclusive.

OCT measurement reproducibility in children is reported to be similar to adults. Additional comparisons of this study to ours are limited because children were not healthy and no segmentation data was provided [28].

Some studies suggest differences between Caucasian, Black and Asian populations [12, 14, 23, 29]. Such differences between ethnicities could not be analysed in our study since all children were Caucasian.

Comparability of our study to the one investigating segmentation data using a custom software and manual segmentation of the layers is further limited because scan parameters differed to the commonly used EDTRS grids. With these settings, the inner retinal layers, which are the focus of our work and seem relevant in both ophthalmologic and neurologic conditions, could not be measured as the area around the fovea investigated was too narrow [13].

Our segmentation data shows that RNFL and IPL in children seem rather comparable to normative values for young adults reported previously [6]. However, the GCL values in our cohort of children are slightly higher. It is described that GCL and IPL decrease with age in adults [6], yet, we cannot determine whether this difference is rather of methodological or biological origin and will need further longitudinal investigations.

## Conclusions

Our study shows that SD-OCT is an effective imaging technique for the examination of the RNFL, TMV and RT, as well as the analysis of individual layers of the retina. We demonstrate feasibility in children from the age of approximately 4 years on.

Normative values comparable to the data presented here do thus far not exist and will be helpful in the clinical use of the standard OCT settings and the system-inherent software. Our data might facilitate diagnosis of ophthalmological and neurological disorders in children in clinical practice and in future studies.

## Supporting information

S1 File Anonymized dataset link: https://doi.org/10.48620/68. (TXT)

## List of abbreviations

ART: automatic real time
ETDRS: early treatment diabetic retinopathy study
GCL: ganglion cell layer; G: global
HIV: human immunodeficiency virus
INL: inner nuclear layer
IPL: inner plexiform layer
N: nasal
NI: nasal inferior
NS: nasal superior
OCT: optical coherence tomography
ONL: outer nuclear layer
OPL: outer plexiform layer
PMB: papillo-macular bundle
pRNFL: peripapillary retinal nerve fibre layer thickness
RT: macular retinal thickness
SD: standard deviation
SD-OCT: spectral domain optical coherence tomography
T: temporal
TI: temporal-inferior
TMV: total macular volume
TS: temporal-superior
